# Hydatidosis of infratemporal fossa with proptosis – an unusual presentation: a case report and review of the literature

**DOI:** 10.1186/s13256-018-1812-y

**Published:** 2018-10-17

**Authors:** Sushma Thapa, Arnab Ghosh, Dilasma Ghartimagar, Supriya Shrestha, Subita Lalchan, O. P. Talwar

**Affiliations:** 1 Department of Pathology, Manipal Teaching Hospital / Manipal College of Medical Sciences, Pokhara, Nepal; 2 Department of Radiology, Manipal Teaching Hospital / Manipal College of Medical Sciences, Pokhara, Nepal

**Keywords:** *Echinococcus*, Hydatid cyst, Infratemporal fossa, Orbit, Proptosis

## Abstract

**Background:**

Hydatid disease is one of the common zoonotic diseases caused by the larval stage of *Echinococcus granulosus.* It is endemic in sheep-raising and cattle-raising areas worldwide and humans are an accidental intermediate host following the ingestion of the larvae. Head and neck involvement of echinococcosis is a rare entity and involvement of the infratemporal region is extremely rare even in endemic areas. Only a few cases of hydatid cysts located in the infratemporal fossa have been reported in the literature. Moreover, extension of the hydatid cyst into the intraorbital region and infiltrating into the surrounding orbital bone is even rarer.

**Case presentation:**

We present a case of a 65-year-old Gurung Nepalese woman with painless proptosis of her left eyeball of 2 months’ duration with recent progressive diminution of vision for 15 days. Radiological findings showed a cystic mass in the left infratemporal fossa extending into the left orbit and involving the surrounding orbital bone. Surgical removal was carried out. On histopathological evaluation, it was reported as hydatid cyst infiltrating into the bone. She was prescribed albendazole and discharged after surgery. However, she was lost to follow up and returned after 15 months with recurrence and proptosis of the same eye. Repeat excision of the lesion was carried out and postoperatively she was administered tablet albendazole. She was found to be disease free after 6 months of follow up.

**Conclusions:**

Clinical and radiological findings are important but may not be sufficient in the preoperative diagnosis of hydatid disease especially if rare sites are involved. Proptosis may be seen in several conditions and orbital or infratemporal hydatidosis, although rare, should be considered a differential diagnosis.

## Background

Hydatid disease or echinococcosis is a zoonosis caused by the larval stage of the genus *Echinococcus* tapeworm, most commonly by *Echinococcus granulosus* and causes worldwide public health and environmental problems [1]. Other forms include *Echinococcus multilocularis*, *Echinococcus vogeli*, and *Echinococcus oligarthrus* [2]. In different studies, the incidence of hydatid disease is found to range from 1 to 220 cases per 100,000 in endemic areas [3, 4]. It is endemic in some parts of the world like the Mediterranean regions, Africa, South America, the Middle East, Australia, and New Zealand [5].

The life cycle of the *E. granulosus* involves a definitive host (dog and other canines) and an intermediate host (usually sheep, cattle, and goats) with humans as accidental hosts following the ingestion of the larvae. Once ingested, the larvae pass into the bloodstream through the intestinal mucosa, where they are most likely to infest the liver because this is the first organ that they pass through [6]. Once the larvae are distributed throughout the intermediate host’s body, they grow into a stage called hydatid cyst [7]. The wall of the cyst is thin and consists of three layers: an outer layer of host origin, a middle cuticle layer, and an inner germinal layer to which are attached brood capsules and scolices. In all sites, hydatid cysts have three layers except for bone; in bone, the hydatid cysts do not have an outer or host layer [8].

Though the liver (60–70%) and lung (20%) are the most common sites for echinococcal disease, nearly any organ can be affected [9–11]. Uncommon sites of involvement include the heart, brain, muscle, salivary glands, spleen, pancreas, bone, adrenals, ovary, and urinary tract [10]. Out of all the different sites, osseous and intraorbital hydatidoses are very rare, accounting for only 0.5–2.5% and < 1% respectively [12, 13]. Head and neck involvement of echinococcosis is a rare entity and involvement of the infratemporal region is extremely rare even in endemic areas. Very few cases of hydatid cysts located in the infratemporal fossa have been reported in the literature [14–17]. Here, we highlight a rare case of primary hydatid cyst of the infratemporal fossa with extension into the orbital region and involving the surrounding orbital bone.

## Case presentation

**Fig. 1:**
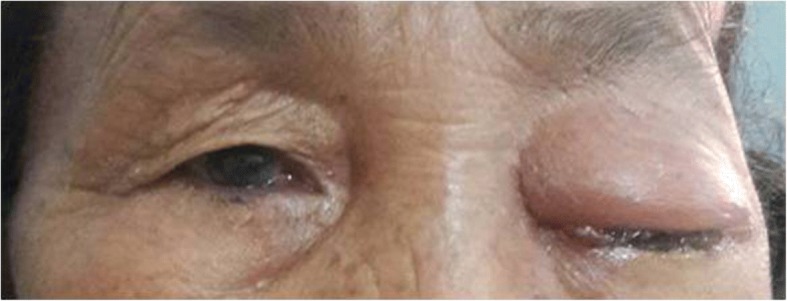
A 65-year-old woman presenting with left-sided proptosis with lid edema

**Fig. 2:**
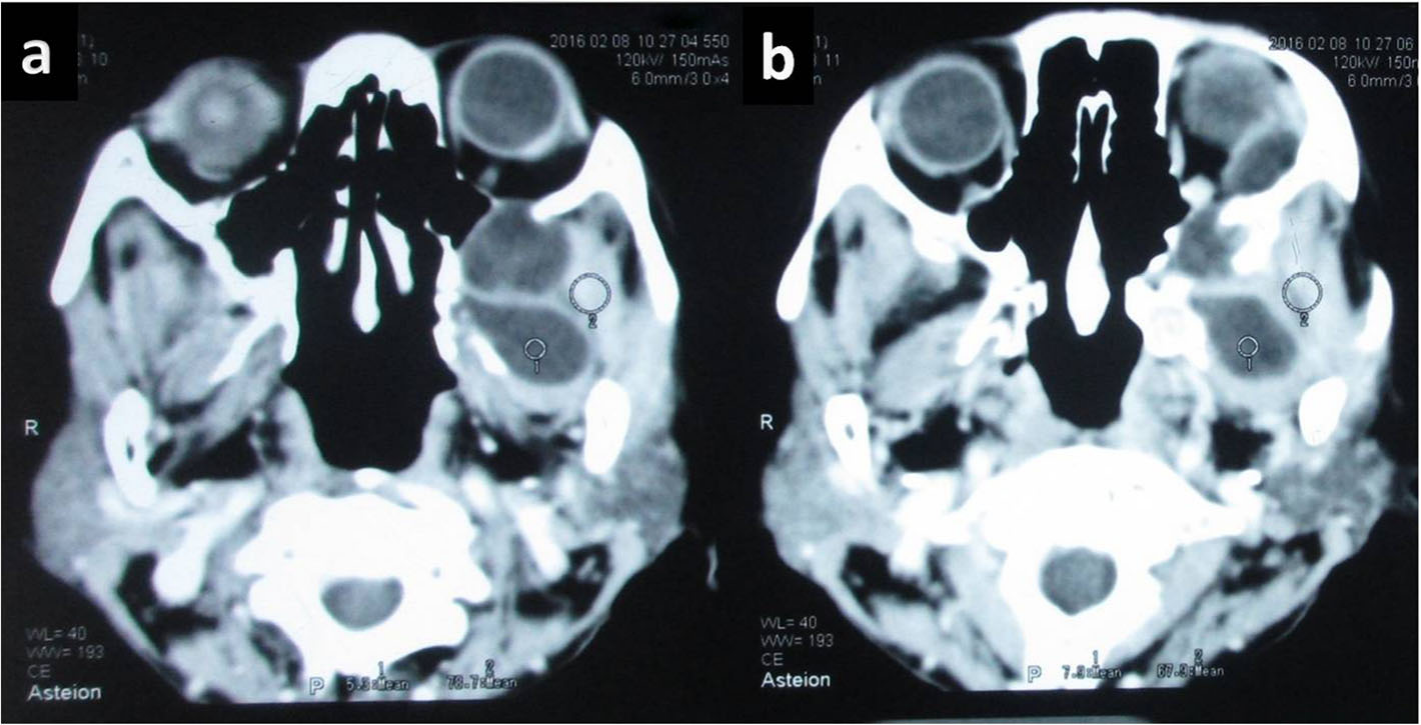
Axial computed tomography images of head and orbit showing multiloculated cystic lesion involving the left infratemporal fossa (**a**) extending to the extraconal space of left orbit (**b**)

**Fig. 3:**
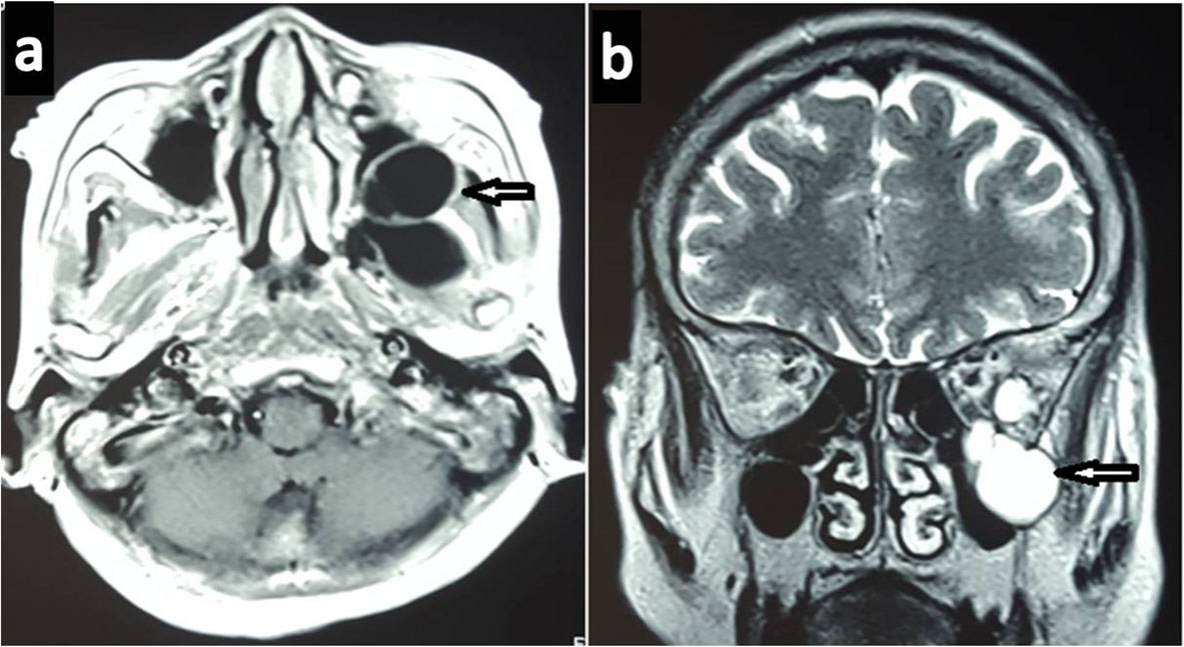
Axial T1-weighted (**a**) and coronal T2-weighted magnetic resonance images showing cystic lesion (*arrows*) within the infratemporal fossa (**b**)

A 65-year-old Gurung Nepalese woman from a remote hilly area, a farmer by occupation, presented with painless bulging of her left eyeball of 2 months’ duration with recent progressive diminution of vision for 15 days. There was no significant family or past medical history. Her general appearance was fair and her Glasgow Coma Scale (GCS) was 15/15. During the admission, her pulse rate was 86 beats/minute, respiratory rate was 24/minute, blood pressure was 100/70 mm Hg, and temperature was 36.9 °C (98.4 °F). There was no lymphadenopathy. On local examination, she had proptosis of her left eye with visual impairment (visual acuity 6/18) but the ocular motility was normal (Fig. 1). The contralateral eye was normal. No other abnormalities were found on neurological examination. A complete blood count showed normal parameters including hemoglobin 110 gm/L, total white blood cell (WBC) count 6.5 × 10^9^/L, total red blood cell (RBC) count 4.25 × 10^12^/L, and total platelet count 399 × 10^9^/L with differential count of 70% neutrophils and 30% lymphocytes. Her urine analysis was within normal limits with 1–2 WBC per high power field and 4–6 epithelial cells per high power field. She had normal renal function test with blood urea and serum creatinine of 4.49 mmol/L and 0.0796 mmol/L, respectively. Her random blood sugar was 5.1 mmol/L. The electrolytes, that is, Na^+^ and K^+^, were 147 and 4.2 mEq/L respectively. Her liver function tests were within normal limits with gamma glutamyl transferase (GGT) of 2.03 μkat/L, total protein of 58 gm/L, albumin of 36 gm/L, globulin of 22 gm/L, albumin to globulin ratio (A:G) ratio of 1.64:1, total bilirubin of 5.13 μmol/L, conjugated bilirubin of 1.71 μmol/L, unconjugated bilirubin of 3.42 μmol/L, aspartate aminotransferase (AST) of 0.53 μkat/L, alanine aminotransferase (ALT) of 0.43 μkat/L, and alkaline phosphatase of 2.03 μkat/L. Routine chest radiography was normal. Computed tomography (CT) of her head and orbit revealed a multiloculated cystic lesion involving the left infratemporal fossa and extending to the extraconal space of left orbit through the infraorbital fissure and causing erosions of left orbital bones (Fig. 2). Magnetic resonance imaging (MRI) showed a well-defined multiloculated cystic lesion within the infratemporal fossa eroding lateral wall of maxillary sinus and floor of the medial cranial fossa. The lesion was hypointense in T1-weighted images and hyperintense in T2-weighted images. The eyeball was displaced antero-medially by the cyst (Fig. 3). Radiological differentials were soft tissue mass and ameloblastoma.

**Fig. 4:**
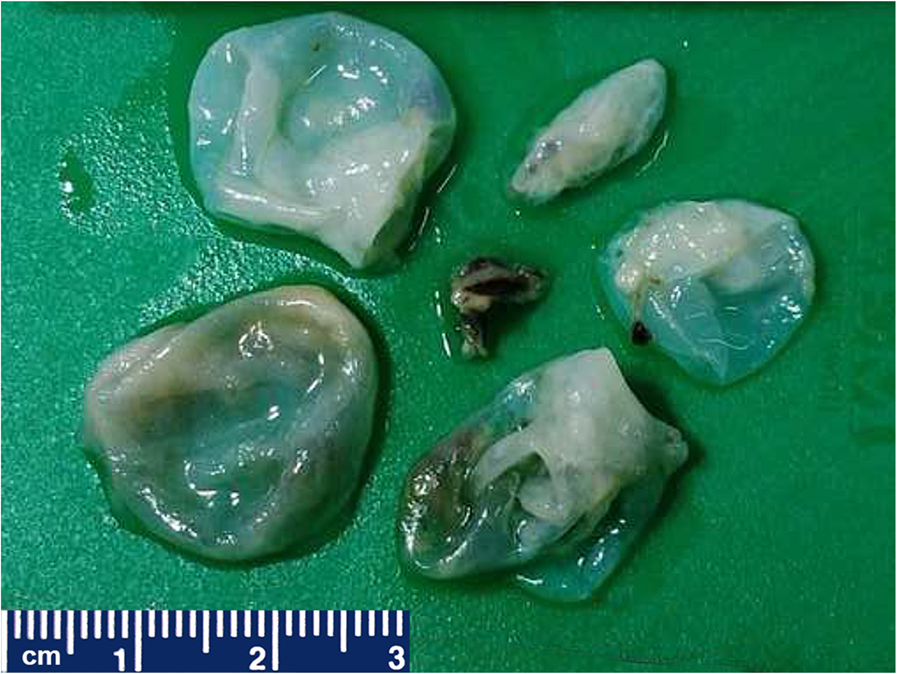
Gross view of multiple translucent cysts filled with clear fluid

**Fig. 5:**
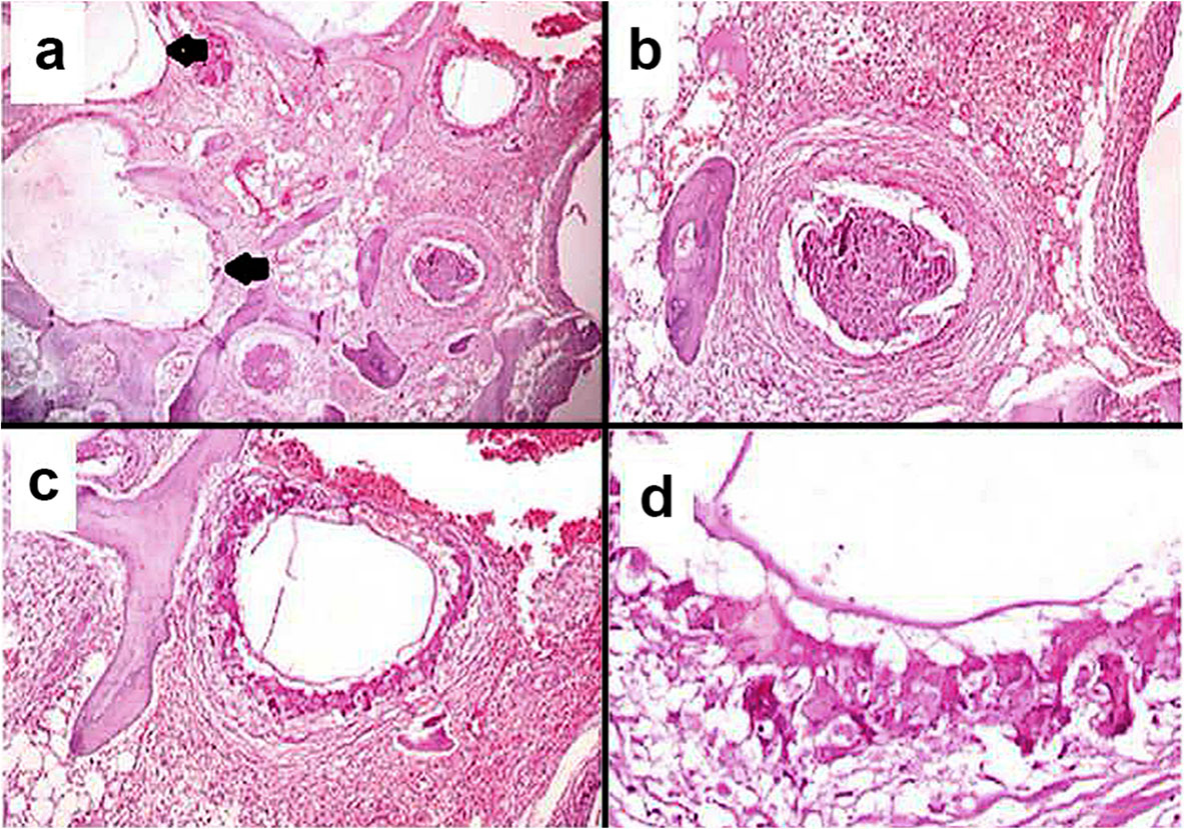
Photomicrograph of hematoxylin and eosin preparations of the specimen shown in Fig. 4 demonstrating acellular laminated eosinophilic structure of the hydatid cyst infiltrating into the bony trabeculae (**a**, 5× objective). The surrounding area showed foreign body giant cell reactions and fibrosis (**b** and **c**, 10× objective; **d**, 20× objective)

She underwent surgery and intraoperatively, multiple cystic lesions eroding the surrounding orbital bones and extending to the infratemporal fossa were noted. Surgical removal of the cysts with left lateral orbitectomy and decompression of the left optic nerve were done. The specimen was received for histopathological examination; it consisted of multiple translucent cysts, the largest measuring 2 × 2 cm and filled with clear fluid (Fig. 4) along with two separate bony bits. The larger bony bit measured 3 × 2 × 1 cm and showed tiny whitish cysts closely attached to and infiltrating into the bone. On microscopy, acellular laminated eosinophilic structures with inner germinal layer were seen infiltrating into the bony trabeculae. The surrounding areas showed foreign body giant cell reaction and fibrosis (Fig. 5). It was reported as hydatid cyst infiltrating into the orbital bone. Ultrasonography (USG) of her abdomen was performed which did not show any visceral involvement. She was advised albendazole on discharge but was lost to follow up. She again presented after 15 months with left orbital swelling. She underwent a CT scan which showed recurrent hydatidosis. On enquiry, she revealed that she did not take oral albendazole as prescribed. No visceral involvement was seen on repeat USG of her abdomen. She underwent repeat surgery with left lateral orbitectomy and decompressive multiloculated cystic lesion in the left infratemporal fossa. After surgery, she was administered tablet albendazole 400 mg twice daily for a period of 28 days. She is now free of disease 6-months postoperatively.

## Discussion

This case report describes a 65-year-old Gurung Nepalese woman with primary hydatid cyst of the infratemporal fossa. The uniqueness of this case is that our patient presented with unilateral ophthalmological signs and symptoms including proptosis due to the extension of the lesion into the orbit and the orbital bones unlike previously reported cases of hydatidosis of the infratemporal fossa where most of the patients presented with swelling of the maxillofacial region [6, 14, 16, 17].

Hydatid disease, caused by the larval stage of *E. granulosus*, is one of the most common zoonotic diseases worldwide and remains a major public health problem in many countries [9]. In addition, the migrating current population is the reason why new cases of hydatid disease are being observed in areas with no previous prevalence [18].

Head and neck involvement of hydatid disease is rare. There have been previous case reports of hydatid cysts in different parts of head and neck area including neck [19–21], nasopharynx [21], skull base [21], maxillary region [22], pterygopalatine fossa [23], and infratemporal fossa [6, 15–17]. Nouroallahian *et al.* reported hydatid cyst of the infratemporal fossa in a 17-year-old female presenting with swelling over the right cheek below the zygomatic process [6]. Pasaoglu *et al*. reported hydatid cyst of the infratemporal fossa in a 9-year-old boy diagnosed by CT scan [16]. Sahin *et al*. reported hydatidosis of the pterygopalatine fossa extending into the infratemporal fossa [14]. Orbital and osseous hydatidoses are rare comprising < 1% and 0.5–2.5% respectively of all cases of hydatid disease [12, 24, 25]. In none of the literatures, has the exact percentage of the involvement of infratemporal fossa been mentioned.

Although a patient’s medical history, family history, occupation, and place of residence may suggest the possibility of hydatid disease, a correct clinical diagnosis is unlikely without a high degree of suspicion of hydatid cysts and radiological findings and, therefore, needs histopathological confirmation [19, 20]. As in other similar case reports [6], the diagnosis of a hydatid cyst in the present case was not considered before the surgery, and a definitive diagnosis was made only by postoperative histopathology.

Clinically, the disease is usually asymptomatic and the clinical symptoms differ depending on the location and pressure effect of the cyst [14]. Most of the reported cases of hydatid cyst in the infratemporal fossa presented with swelling in the maxillofacial region [6, 14, 16, 26]. In the present case, although the main cyst was in the infratemporal fossa, the patient presented with an ophthalmological complaint due to the extension of the cyst into the orbital region causing pressure symptoms. The ocular symptoms of unilateral exophthalmos may be present in other orbital diseases such as retinoblastoma, capillary hemangioma, glioma, and lymphangioma. So the clinical diagnosis of hydatid cyst with orbital symptoms is often difficult [27].

Different serological tests such as Casoni’s intradermal test, complement fixation test, and ELISA may also be helpful for the diagnosis of hydatid disease but are not very reliable [14, 24]. In our case, serological tests were not available. Radiological findings are usually more important in preoperative diagnosis. On ultrasound, diagnostic “double layer sign” of the cyst wall, “spoked wheel pattern,” and “water lily sign” are typically described in the literature [24]. These cysts may appear as well-defined, unilocular or multilocular cysts on a CT scan and as mildly enhancing cystic lesions that have fluid attenuation with few calcifications and multiple septae on a contrast-enhanced computed tomography (CECT) scan [24]. MRI shows a well-defined T1 hypointense and T2 hyperintense cystic lesion [27]. In this case, the preoperative radiological examination of the mass could not indicate the right diagnosis as the lesion was destroying and infiltrating into the surrounding orbital bones. The diagnosis was confirmed by histopathological examination of the lesion demonstrating an eosinophilic acellular laminated structure with inner germinal layer infiltrating in between the bony trabeculae.

Molecular studies show that *E. granulosus* exhibit substantial genetic diversity that has important implications for the design and development of vaccines. Deoxyribonucleic acid (DNA) approaches, like the use of DNA probes, are useful for the accurate identification of this genus [1].

Definitive treatment for hydatid cyst is surgical removal of the cyst which remains the gold standard, although particular efforts should be made to prevent the breakage of the cyst during the procedure, which could lead to anaphylaxis and/or cyst recurrence [6]. The prognosis is excellent in hydatidosis when treated by total removal of the cyst without rupture [28]. Besides surgery, non-conventional treatment like puncture, aspiration, injection, and re-aspiration (PAIR) had been studied recently and was found safe and effective [1]. In medical treatment, the imidazole group of drugs (mebendazole and albendazole) is widely used but is contraindicated in pregnancy, and in hepatic and renal impairment [1].

## Conclusions

Clinical and radiological findings are important but may not be sufficient in the preoperative diagnosis of hydatid disease especially if rare sites are involved which may necessitate histopathological confirmation. Hydatidosis of infratemporal fossa extending into the orbit is a very rare entity. It should be considered a differential diagnosis of any swelling of the head and neck region especially in areas with high prevalence.

## Abbreviations

A:G: Albumin to globulin ratio
ALT: Alanine aminotransferase
AST: Aspartate aminotransferase
CECT: Contrast-enhanced computed tomography
CT: Computed tomography
GCS: Glasgow Coma Scale
GGT: Gamma glutamyl transferase
MRI: Magnetic resonance imaging
PAIR: Puncture, aspiration, injection, and re-aspiration
RBC: Red blood cell
USG: Ultrasonography
WBC: White blood cell
